# Fidgetin-like 2 knockdown increases acute neuroinflammation and improves recovery in a rat model of spinal cord injury

**DOI:** 10.1186/s12974-025-03344-3

**Published:** 2025-03-10

**Authors:** Austin N. Smith, Samantha Nagrabski, Lisa Baker, Adam H. Kramer, David J. Sharp, Kimberly R. Byrnes

**Affiliations:** 1 Neuroscience Program, Uniformed Services University of the Health Sciences, Bethesda, MD USA; 2 Department of Anatomy, Physiology and Genetics, Uniformed Services University of the Health Sciences, Bethesda, MD USA; 3 MicroCures, Inc., New York, NY USA; 4 Department of Molecular Pharmacology, Albert Einstein College of Medicine, Bronx, NY USA

**Keywords:** Cytoskeleton, Fignl2, Gene therapy, Inflammation, Macrophages, Microglia, Nanoparticle siRNA, Neurorestoration

## Abstract

Spinal cord injury (SCI) can cause permanent dysfunction proceeding from multifaceted neuroinflammatory processes that contribute to damage and repair. Fidgetin-like 2 (FL2), a microtubule-severing enzyme that negatively regulates axon growth, microglial functions, and wound healing, has emerged as a potential therapeutic target for central nervous system injuries and neuroinflammation. To test the hypothesis that FL2 knockdown increases acute neuroinflammation and improves recovery after SCI, we examined the effects of nanoparticle-encapsulated FL2 siRNA treatment after a moderate contusion SCI in rats. SCI significantly increased FL2 expression in the lesion site and rostral to the lesion 1 day post-injury (dpi). A single treatment of FL2 siRNA after injury led to modestly improved locomotor recovery consistent with the preservation of corticospinal tract function, accompanied by reduced inflammation and increased presence of oligodendrocytes. In determining the acute effects of treatment, RNA sequencing and gene set enrichment analyses revealed that FL2 siRNA modulates early cellular responses, including chemokine signaling, both pro- and anti-inflammatory immune reactions, and neurotransmitter signaling pathways at 1, 4, and 7 dpi. Follow-up analyses at 4 dpi using dual in situ hybridization and immunohistochemistry demonstrated that SCI increased FL2 mRNA and that FL2 was colocalized with microglia/macrophages. FL2 downregulation resulted in a marked accumulation of microglia at the lesion site, accompanied by increased inflammatory markers (IL-1β, TGF-β1, and CD68). The results suggest SCI induces an increase in FL2 expression that undermines acute inflammatory responses as well as spinal cord integrity and growth. Overall, our study suggests that targeting FL2 holds promise as a therapeutic strategy for treating SCI.

**Supplementary Information:**

The online version contains supplementary material available at 10.1186/s12974-025-03344-3.

## Introduction

Spinal cord injury (SCI) can cause permanent motor, sensory, and autonomic dysfunction. While there have been significant advancements in understanding SCI mechanisms and pathophysiology, treatment options for clinical application remain limited [1]. This may be due to the variability in primary injuries and the complexity of secondary injuries, which involve cell death cascades, excitotoxicity, inflammation, oxidative stress, and other sequelae of the initial trauma [2, 3]. This continuation of injury begins within minutes of an SCI and may persist for months or years. Therapies aimed at influencing secondary injury have shown the potential to limit damage progression and improve long-term outcomes [4].

During secondary injury, glia and infiltrating peripheral cells respond to prevent the spread of damage and restore homeostasis. They can also establish an unfavorable or growth-inhibitory microenvironment [5]. At the same time, neurons and oligodendrocyte lineage cells are trying to survive and regrow [6, 7]. Optimizing cellular responses during the acute stage may improve wound healing and recovery after SCI [8].

Immune responses following injury to the central nervous system (CNS), are both beneficial and detrimental to SCI [9, 10]. This is particularly true for microglia, the resident immune cells of the CNS. The acute microglial response can be beneficial for long-term outcomes [11–15], while the delayed response is associated with chronic microglial activation and systemic inflammation [16–18]. Research suggests that insufficient resolution of early inflammation can limit CNS wound healing and cause inflammation to remain in a non-resolving state or “resolution plateau” [19–23]. However, research shows that a well-regulated neuroimmune response, even when activated to a greater extent, can improve long-term outcomes [11, 24, 25].

The microtubule regulatory protein fidgetin-like 2 (FL2) is a therapeutic target to promote wound healing. FL2 is a microtubule-severing enzyme that negatively regulates cell movement and growth [26, 27]. The enzyme localizes to the leading edge of migrating cells and is proposed to suppress cell movement by paring down labile microtubules near the cortex: in vitro, knockdown of the enzyme in osteosarcoma cells led to a twofold increase in the velocity of cell motility, increased the density of dynamic microtubules, and decreased RhoA activation at the leading edge. In regenerating neurons, FL2 appears to similarly suppress the advancement of growth cones, with depletion of FL2 from dissociated adult dorsal root ganglion neurons enhancing the rate of neurite regeneration and increasing the ratio of dynamic to total microtubules in the distal-most region of neurites [28].

FL2-targeting therapies show efficacy in improving the rate and quality of healing after cutaneous and corneal injuries [26, 29, 30]. More importantly, FL2 siRNA treatment improves functional regeneration after peripheral nerve crush and transection injuries [28]. Existing data suggests that FL2 regulates CNS development [31], and recent research found that inflammatory stimuli reduce microglial expression of FL2 [32]. Furthermore, FL2 siRNA increases microglial motility, phagocytic activity, and certain cytokine secretion in vitro [32]. This research suggests that FL2 is a therapeutic target for CNS traumatic injuries. However, the role of FL2 in the context of CNS injury has not been characterized, including its effects on neuroinflammatory responses.

Based on prior research, we hypothesized that FL2 siRNA treatment would increase the beneficial inflammatory response and improve recovery after SCI. While prior research has demonstrated that FL2 is a therapeutic target to promote wound healing and regulate CNS cell types, no study to date has examined FL2 in a SCI injury model. Therefore, this study examined FL2 after a moderate contusion SCI in rats. We evaluated the effects of FL2 knockdown on the acute neuroinflammatory response and long-term recovery after SCI.

## Materials and methods

### Subjects

Young adult (3–4 months) male Sprague-Dawley rats (Taconic, Germantown, MD) were used for all experiments. A total of 150 rats (332 g ± 64 g) were included in this study. Rats were dual-housed with *ad libitum* access to food and water under a 12-hour light/dark cycle. All animal procedures were approved by the Uniformed Services University IACUC and complied fully with the principles set forth in the “Guide for the Care and Use of Laboratory Animals.” Rats were randomly assigned to surgery and treatment groups.

### Contusion spinal cord injury

Rats were anesthetized with isoflurane (4% induction, 2% maintenance in 50% O_2_) and placed on isothermal heating pads warmed to 37 °C with rectal thermometer feedback for temperature maintenance. A laminectomy was performed at the ninth thoracic vertebra (T9) to expose the spinal cord. A moderate injury (200 kilodynes force) was induced with the Infinite Horizon Impactor (Precision Systems Incorporated, Natick, MA), resulting in a reproducible decline in hindlimb locomotive ability [33]. Sham animals received anesthesia and a laminectomy without an impact. Naive animals did not undergo any surgery or receive any treatment.

Postsurgical procedures included housing on soft bedding and analgesic (acetaminophen, 200 mg/kg) in drinking water for 48 h. Because rats lose the micturition reflex [34], bladders were manually expressed twice daily until micturition returned. Topical antibiotics and subcutaneous antibiotics (Baytril) were administered as necessary if the rats showed signs of infection.

### siRNA nanoparticle synthesis and administration

The siRNA nanoparticles were synthesized as previously described to protect the siRNA from enzymatic degradation during in vivo delivery [28, 32]: 500 µL of tetramethyl orthosilicate (TMOS) was hydrolyzed with 100 µL of 1 mM HCl by sonication on ice for 15 min. TMOS (100 µL) was added to 900 µL of 10 µM of siRNA containing 10 mM phosphate, pH 7.4, and then allowed to solidify into a block gel at room temperature (10–15 min). The metal-oxo skeleton formed by the TMOS entraps the siRNA. The block gel was frozen at − 80 °C and then lyophilized overnight. The silicate particles were ground with a mortar and pestle to ensure any larger pieces were further broken up. The lyophilized nanoparticles were then resuspended in PBS and stored at − 80 °C until use. The siRNAs included pooled rat FL2 (siRNA from MilliporeSigma: SASI_Rn02 00314854, target sequence: CTGGATGTCTCCTCCACCA; SASI_Rn02 00314855, target sequence: CAGAGGATGGGACCGGCAA; SASI_Rn02_ 00389576, target sequence: CCTCCAACCTCCTCAAGAG) or the negative control siRNA (MilliporeSigma, Universal Negative control B).

Immediately after injury or sham surgeries, rats received an intrathecal injection of 10 µl of 10 µM SiCon or SiFi2 into the lesion epicenter (T9), followed by loading of 100 µl into the epidural space. Treatments were administered by an investigator blinded to the treatment identity.

### Functional analysis

The Basso Beattie Bresnahan (BBB) locomotor scale was used to assess hindlimb function after SCI [35]. Locomotor behavior was evaluated on the first postoperative day and then once a week up to 28 days post-injury (dpi). Rats were placed in an open field apparatus and encouraged to locomote continuously without crawling up the sides of the enclosure for five minutes. At least two examiners blinded to the treatments recorded the motor performance of left and right hindlimbs on a scale from 0 (no movement) to 21 (normal locomotion). The lower score was recorded when behavior was borderline, or examiners disagreed. Scores from the left and right limbs were averaged to obtain the total BBB score. Injured rats with a score below five and sham rats with a score above twenty at 1 dpi met inclusion criteria. The final sample sizes for each experiment are provided in Table 1.

**Table 1:** Overview of experimental group sizes including surgery, treatment, time point, and outcome measures

**FL2 Characterization Experiments**						
Naive	Sham	1 dpi	7 dpi	14 dpi	30 dpi	90 dpi
3P	5P	6P	5P	6P	9P	9P
Excluded	2 (did not meet inclusion criteria)					

**FL2 siRNA Treatment Experiments**								
Surgery	Time Point & Treatment							
	1 dpi		4 dpi		7 dpi		28 dpi	
	SiCon	SiFi2	SiCon	SiFi2	SiCon	SiFi2	SiCon	SiFi2
Injury	5R	5R	5R, 5H	5R, 5H	5R	5R	12F, 6H	12F, 6H
Sham	4R	5R	4R, 5H	4R, 5H	4R	4R		
Naive							8F	
Excluded	1 (did not meet inclusion criteria)		6 (2 surgery complication, 4 did not meet inclusion criteria)		2 (surgery complication)		4 (2 mortality after surgery, 1 did not meet inclusion criteria, 1 infection)	

# = Sample size, P = Polymerase Chain Reaction, R = RNA sequencing, H = Histology, F = Function

### Tissue collection

Spinal cord tissue was collected between 1 and 90 dpi. At each time point, rats were anesthetized with an intraperitoneal injection of Euthasol (pentobarbital sodium and phenytoin sodium; 200 mg/kg) and intracardially perfused with saline. Fresh frozen tissue was flash-frozen on dry ice and stored at − 80 °C. For fixed tissue, saline was followed by 10% buffered formalin. Spinal cord tissue was dissected in 5 mm segments, 2.5 mm caudal and 2.5 mm rostral to the lesion epicenter or laminectomy site, which was identifiable by bruising, scar tissue, or missing vertebral bone. The tissue was post-fixed for 24 h in 10% buffered formalin and cryoprotected in 30% sucrose at 4 °C.

### Quantitative real-time polymerase chain reaction (qPCR)

Samples were homogenized using a Bullet Blender (Next Advance), and the RNA was extracted using TRIzol (Fisher, #11596026). RNA concentration and quality were measured using a nanodrop, and the extracts were stored at − 80 °C. RNA was reverse transcribed using the SuperScript IV VILO kit (Invitrogen, #11766050), following the SS IV VILO protocol but with a reverse transcription temperature of 65 °C instead of 50 °C (as is recommended for GC-rich targets). Power Sybr green Master Mix was used for qPCR using the 7300 Real-Time PCR system (Applied Biosystems). *Prkg1* was used as the housekeeping gene for normalization. The following primer sequences were used: *Fignl2* (GAGTTGGCTGCAGTGTGAATG and CTCTGTGCTTCTGTCTCTGT﻿) and *Prkg1* (TGACGTTCGCTGTTCTTACATC and GAAGACTCACCAAGTGAAGACC). Results were analyzed using the ddCT method. For samples that were included in more than one qPCR run, the RQs from each run were averaged for the final analysis.

### Gait analysis

At 21 dpi, gait locomotion was analyzed using DigiGait™ Imaging System (Mouse Specifics, Inc., Framingham, MA, USA). Rats were placed in the testing chamber with a motorized, transparent treadmill belt and allowed to acclimate. The treadmill speed was set to 15 cm/s. The trial was stopped when three seconds of constant stepping were recorded. Animals that could not achieve three seconds of walking were excluded from analyses (4 of 12 excluded per group). The videos were analyzed with the DigiGait™ software to evaluate paw angle (paw placement relative to the long axis of the animal in motion). Injury groups were also compared to naive controls.

### Immunohistochemistry

Standard immunohistochemistry was performed on fixed spinal cord segments sectioned longitudinally at 15 μm thickness. Sections were incubated with primary antibodies overnight at 4 °C. The full list of antibodies can be found in Supplementary Table S1. Sections were then incubated with Alexa Flour secondary antibodies (1:1000, Invitrogen) for 1 h at room temperature and coverslipped with HardSet Mounting Media with DAPI counterstain for nuclei (Vectashield). Five sections were analyzed per animal at even intervals throughout the spinal cord. Images were captured at the lesion site or in the penumbra using a fluorescence microscope (Olympus) at 20X magnification. ImageJ was used to quantify immunostaining as pixel density, area, or cell count.

### RNA sequencing

Fresh frozen spinal cord tissue was dissected in 5 mm segments from the lesion or laminectomy site at 1, 4, or 7 dpi. Then, samples were submitted to Azenta Life Sciences (Chelmsford, Massachusetts) for RNA extraction and sequencing. RNA was sequenced on an Illumina Nova Seq Xplus (20 million paired-end reads per sample). The quality of sequencing output was assessed using FAST-QC. Sequence reads were trimmed using Trimmomatic v.0.36. Trimmed reads were mapped to the Rattus norvegicus Rnor6.0 reference genome using the STAR aligner v.2.5.2b. Unique gene hit counts were calculated by using featureCounts from the Subread package v.1.5.2.

### Transcriptome analysis

RNA-seq analyses were performed using R (v4.4.0). Gene Set Enrichment Analysis (GSEA) of RNA-seq data was performed with the org.Rn.eg.db and clusterProfiler packages. The Kyoto Encyclopedia Gene and Genomics (KEGG) database was used as a priori-defined genomic pathways. For each pathway, GSEA calculated the degree to which genes in each pathway are overrepresented at the top or bottom of the entire list of genes ranked by fold change. The results are enrichment scores, the maximum deviation from zero. Enrichment scores were used to create a permutation-generated null distribution, from which the significance of enrichment scores is calculated. Pathways with an adjusted *p* value < 0.05 were considered significantly enriched KEGG pathways. Normalized enrichment scores were calculated to account for differences in gene set sizes. GSEA data was visualized using ggplot.

Differential gene expression analysis was performed on the RNA sequencing gene count data using DESeq2. The Wald test was used to generate *p* values and log2 fold changes. Genes with an adjusted *p* value < 0.05 and absolute log2 fold change (log2FC) > 1 were considered differentially expressed genes for each comparison.

### Dual in situ hybridization and immunohistochemistry

RNAscope™ in situ hybridization was performed on fixed tissue sections per the manufacturer’s instructions according to the Multiplex Fluorescent Reagent Kit v2 (ACD, #UM323100). Probes were used to detect mRNA expression of FL2 (ACD, #1247681-C2). Immediately after amplification, signal development, and blocking, the sections were incubated with rabbit anti-Iba1 (1:250) in Co-Detection Antibody Diluent (ACD, #323160) overnight at 4 °C. Sections were incubated with Alexa Flour secondary antibodies (1:1000, Invitrogen) for 1 h at room temperature before a 30-second incubation with drops of RNAscope Multiplex FL v2 DAPI. Slides were coverslipped with ProLong™ Gold antifade reagent (Invitrogen, #P36930). Five images (randomly obtained from the lesion center and surrounding four quadrants) of each tissue section were captured using a fluorescence microscope (Olympus) at 20X magnification. QuPath software v0.5.1 [36] was used to quantify the puncta of FL2.

### Statistical analysis

Statistical analysis was performed in GraphPad Prism v10.2.0. As appropriate, histological and functional data were analyzed using unpaired *t*-test, the Brown-Forsythe and Welch ANOVA, ordinary one-way ANOVA, and two-way ANOVA. The Greenhouse–Geisser correction was used in repeated measures ANOVA. Fisher’s least significant difference test was performed as a global test to preserve the experiment-wise type I error. Dunnett’s test was used to perform multiple comparisons when there was unequal size or variance; otherwise, the Šidák correction was used. A *p* value < 0.05 was considered statistically significant. All data are presented as mean ± standard error of the mean (SEM).

## Results

### Spinal cord injury increases FL2 expression

**Fig. 1:**
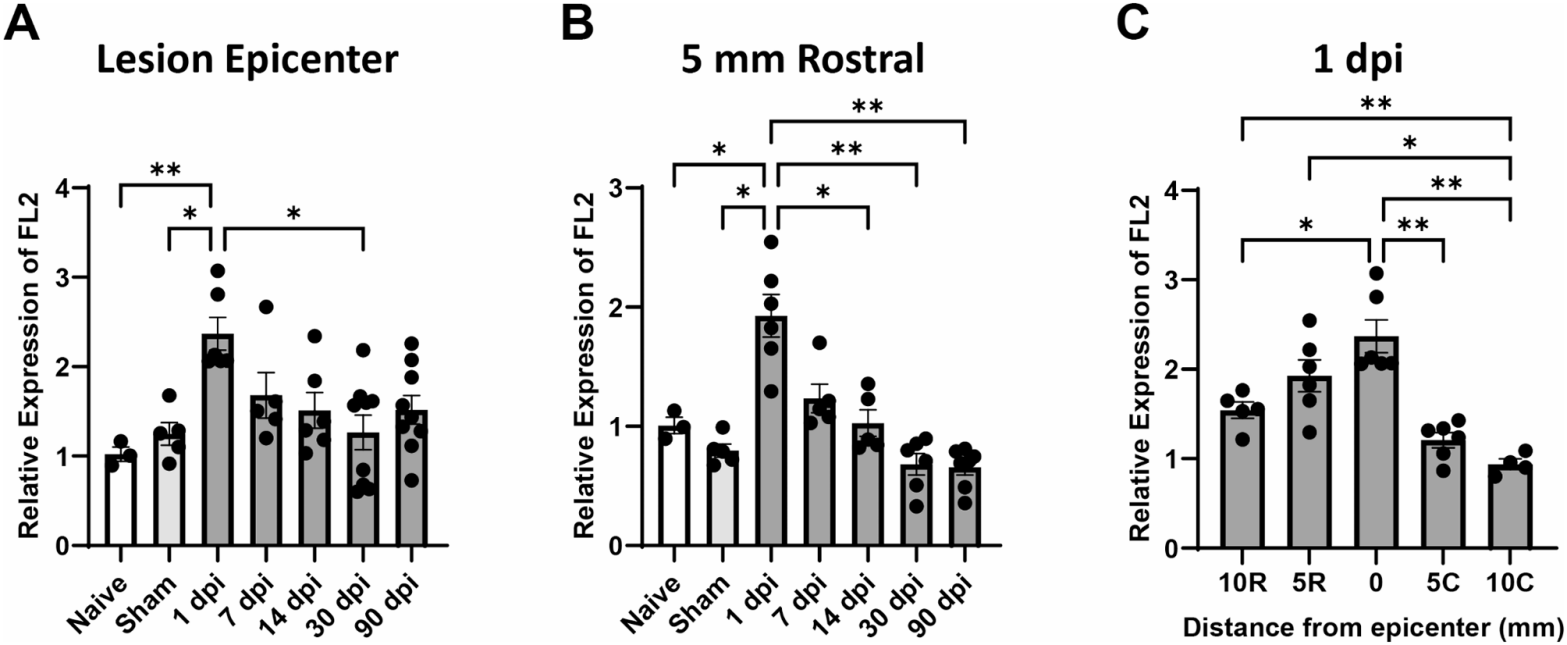
FL2 mRNA levels are increased at the lesion and directly rostral to the lesion after SCI. Relative quantities of *Fignl2* (FL2) mRNA at the T9 lesion site (**A**) and 5 mm rostral to the lesion (**B**) over time after contusion spinal cord injury. The samples were normalized to naive samples from 3 animals at the same spinal cord level. (**C**) FL2 levels 1 dpi plotted by distance rostral (R) or caudal (**C**) to the epicenter of the lesion. FL2 mRNA levels were quantified using the ddCT method and normalized to the housekeeping gene *Prkg1*. *N* = 3–9/group. Brown-Forsythe and Welch ANOVA with Dunnett’s multiple comparison tests. *p < 0.05, **p < 0.01. Bars represent mean ± SEM

FL2 mRNA levels in spinal cord samples were quantified with RT-qPCR to assess temporal and spatial expression changes. SCI increased the expression of FL2 in the 5 mm segment of the lesion site (Fig. 1A; *p* = 0.001, Welch’s ANOVA test with Dunnett’s T3 *post hoc* tests). FL2 expression was significantly increased in the lesion as early as 1 dpi compared to naive (*p* = 0.007) and sham (*p* = 0.02). There were no significant differences in FL2 expression compared to naive or sham between 7 and 90 dpi. In comparison to the elevated FL2 levels at 1 dpi, FL2 expression was significantly reduced at 30 dpi (*p* = 0.02). A similar pattern of significant differences in FL2 expression was found in the 5 mm segment rostral to the lesion segment (Fig. 1B; *p* < 0.001, Welch’s ANOVA test with Dunnett’s T3 *post hoc* tests). FL2 expression was significantly increased rostral to the lesion at 1 dpi compared to naive (*p* = 0.04) and sham (*p* = 0.01). In comparison to the elevated FL2 levels at 1 dpi, FL2 expression was significantly reduced at 14 dpi (*p* = 0.04), 30 dpi (*p* = 0.007), and 90 dpi (*p* = 0.007). No significant increases in FL2 expression were observed 5 mm caudal to the lesion at 1 dpi or 10 mm rostral and caudal between 1 and 90 dpi (Supplementary Figure S1).

To further assess the spatial expression of FL2 at 1 dpi, mRNA levels were compared between the different segments of the injured spinal cord. There were significant differences among spinal cord segments (Fig. 1C; *p* < 0.0001, Welch’s ANOVA test with Dunnett’s T3 *post hoc* tests), showing that FL2 was increased in the lesion site compared to 10 mm rostral (*p* = 0.04), 5 mm caudal (*p* = 0.006), and 10 mm caudal (*p* = 0.002). FL2 expression was also increased at 5 mm rostral (*p* = 0.01) and 10 mm rostral (*p* = 0.007) compared to 10 mm caudal. These results indicate that FL2 expression is acutely upregulated after SCI in the lesion and immediately rostral to the lesion.

### Effects of FL2 siRNA on locomotor recovery after SCI

**Fig. 2:**
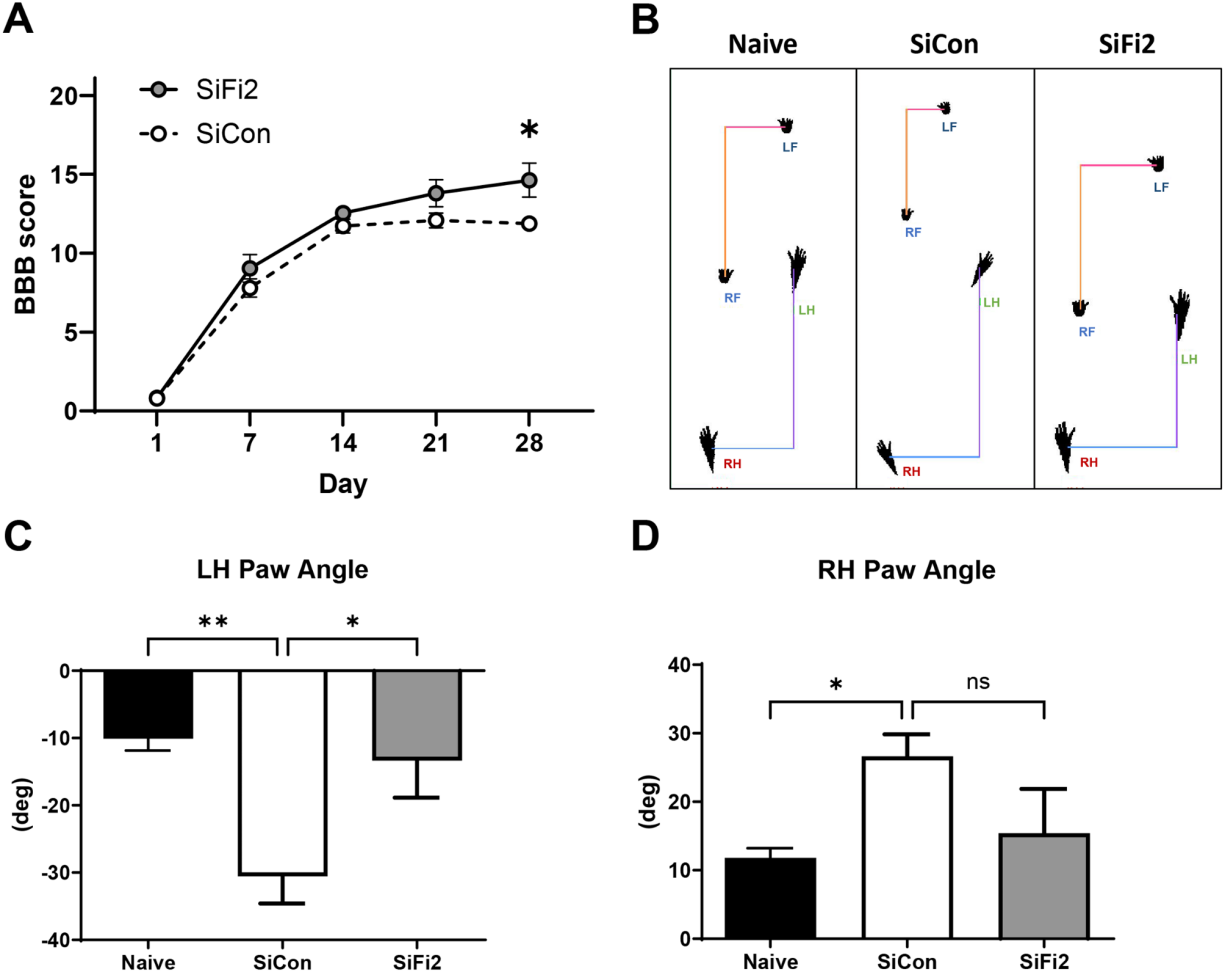
FL2 siRNA (SiFi2) improved functional recovery after SCI. (**A**) Time course of BBB locomotor scale scores following contusion spinal cord injury. FL2-siRNA (SiFi2) and non-targeting siRNA (SiCon) show significant recovery compared to 1 dpi. SiFi2 increased BBB score compared to SiCon. *N* = 12/group, repeated measures two-way ANOVA with Šídák multiple comparison tests. (**B**) Representative footprint analysis using the DigiGait™ gait analysis. (**C**) Left hindlimb (LH) and (**D**) right hindlimb (RH) paw angle measurements. SiCon rats exhibited more rotation compared to naive animals whereas SiFi2 returned paw angle to naive values, suggesting a restoral of corticospinal tract control of the hind paws. *N* = 8/group. One-way ANOVA with Dunnett’s multiple comparison tests. *p < 0.05, **p < 0.01, ns = not significant. Bars represent mean ± SEM

In order to determine whether FL2 knockdown has a therapeutic effect on functional recovery after SCI, rats received nanoparticle-encapsulated FL2 siRNA (SiFi2) or negative control siRNA (SiCon) administered immediately after SCI before undergoing functional assessments up to 28 dpi. Hindlimb locomotor function was evaluated weekly with the BBB locomotor grading scale. All injured animals exhibited hindlimb paralysis (BBB < 5) at 1 dpi. Over the course of recovery, SiFi2 improved gross locomotor skills, with a significant main effect after SCI in comparison to SiCon (Fig. 2A, *p* = 0.046, two-way ANOVA with Šídák multiple comparison tests.). SiFi2 treatment significantly increased BBB score at 28 dpi (*p* = 0.03) with an average 2.75 point locomotor improvement compared to SiCon.

Gait analysis was performed with DigiGait™ to quantitatively assess locomotor kinematics at 21 dpi derived from digital footprints (Fig. 2B). Results showed a significant effect on paw angle with SCI and siRNA administration (Fig. 2C, *p* = 0.02, one-way ANOVA with Dunnett *post hoc* tests). SiCon rats demonstrated impaired paw rotation after SCI with significantly externalized left hind paw angle by − 20 degrees on average compared to naive rats (*p* = 0.004). SiFi2 rats showed significant improvement of the left hind paw angle, returning to within the range of paw angles exhibited by the naive rats (*p* = 0.01). SiCon rats exhibited similar externalized right hind paw angles compared to naive rats (Fig. 2D, *p* = 0.04, one-way ANOVA with Dunnett *post hoc* tests), but improvement with SiFi2 did not reach statistical significance (*p* = 0.07). These results suggest that SiFi2 promoted corticospinal tract (CST) control of the hind paw rotation [37].

### FL2 siRNA reduces chronic microglial presence and increases oligodendrocytes

**Fig. 3:**
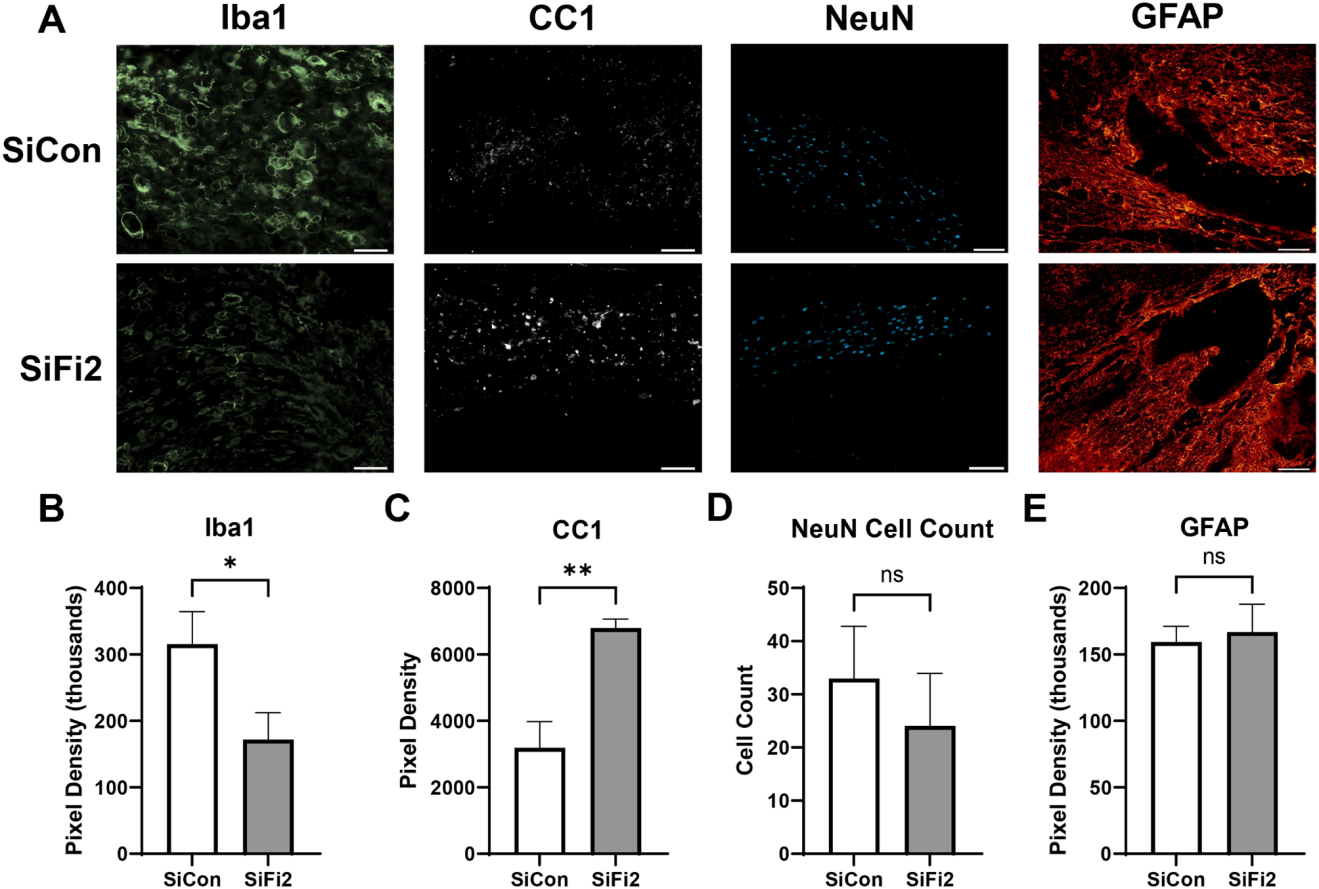
FL2 siRNA significantly alters microglia and oligodendrocytes in the lesion after SCI. (**A**) Representative immunofluorescence images of Iba1 (microglia, green), CC1 (oligodendrocytes, white), NeuN (neurons, blue), and GFAP (astrocytes, red) in the lesion epicenter (Iba1, GFAP) or in the penumbra (CC1, NeuN) at 28 dpi after SCI with siRNA (SiCon) or FL2 siRNA (SiFi2) treatment (20X; scale bar = 50 μm). Quantification of pixel density of Iba1 (**B**) and CC1 (**C**) shows that SiFi2 altered the response, with a significant reduction in Iba1 and a significant increase in CC1. Count of NeuN positive cells (**D**) or GFAP pixel density (E) did not show a statistically significant difference between groups. *N* = 5/group. Unpaired *t*-tests. *p < 0.05, **p < 0.01, ns = not significant. Bars represent mean ± SEM

To determine if acute FL2 downregulation resulted in chronic changes in histopathology at the lesion site, immunohistochemistry was performed at 28 dpi (Fig. 3A). Immunostaining for microglia (Iba1) and oligodendrocytes (CC1) showed significant changes, with reduced Iba1 and increased CC1 immunopositive pixel density at the lesion epicenter after SiFi2 treatment (Fig. 3B–C; *p* = 0.027, *p* = 0.002, respectively, unpaired *t*-tests). Quantitation of number of neurons (NeuN) in the perilesion gray matter or astrocytes (GFAP) in the scar surrounding the lesion showed no significant difference between groups (Fig. 3D, E; *p* = 0.27, *p* = 0.45, respectively, unpaired *t*-test).

### SCI and FL2 siRNA effects on acute gene expression

GSEA was conducted to identify functional gene pathways that may explain the long-term improvements after acute FL2 downregulation. KEGG pathway enrichment was analyzed at 1, 4, and 7 dpi for the following comparisons: Sham + SiCon vs. SCI + SiCon, Sham + SiCon vs. Sham + SiFi2, and SCI + SiCon vs. SCI + SiFi2. The complete lists of significant KEGG pathways resulting from analyses are available in Supplementary Materials (Supplementary Tables S2–S10).

SCI induced many significantly enriched KEGG pathways, as shown in comparisons between SCI + SiCon compared to Sham + SiCon (Supplementary Figure S2). There were 60, 60, and 63 significant gene sets at 1, 4, and 7 dpi, respectively. Upregulated pathways included immune responses (cytokine-cytokine receptor interaction, hematopoietic cell lineage, and chemokine signaling) and cell death (apoptosis, p53 signaling). Downregulated pathways included neuronal function (neuroactive ligand-receptor interaction, long-term potentiation, long-term depression) and mitochondrial respiration (oxidative phosphorylation, Parkinson’s disease).

**Fig. 4:**
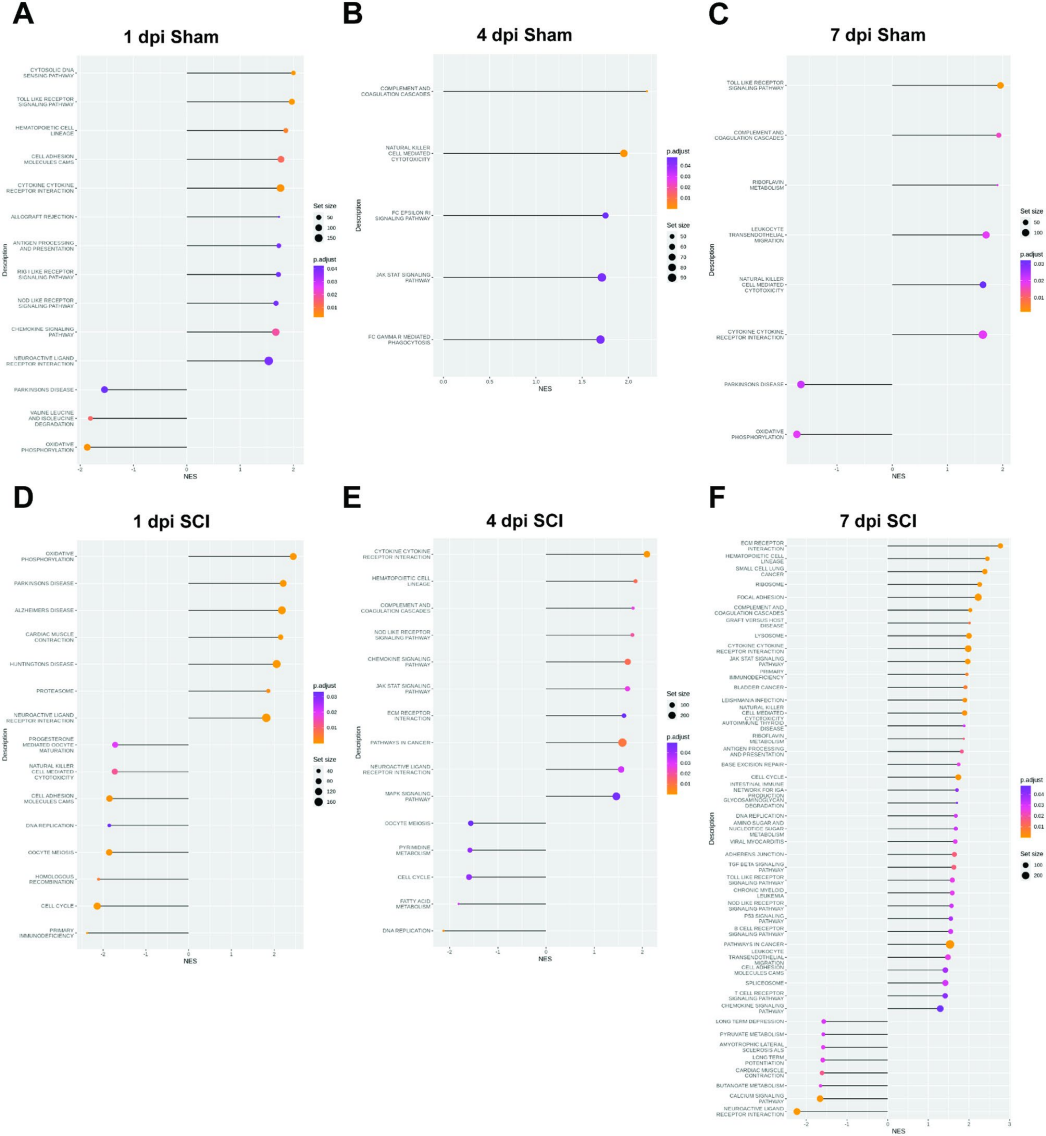
Gene set enrichment analysis (GSEA) of all genes in the spinal cord with FL2 siRNA. Lollipop plots depicting statistically significant gene pathways identified in GSEA comparing control siRNA (SiCon) and FL2 siRNA (SiFi2) treatment in the acute stage after sham (**A**–**C**) or SCI (**D**–**F**). The X axis shows the normalized enrichment score (NES), which indicates the degree to which genes in a particular set were upregulated (positive) or downregulated (negative) while accounting for the size of each gene set. The Y axis lists the individual KEGG pathways. The dot size is proportional to the total number of genes within each pathway (Set size). The dot color displays the range of adjusted *p* values < 0.05

GSEA identified significantly enriched pathways in groups that received SiFi2 at all time points compared to groups that underwent the same injury condition and received SiCon. Significant results from 1, 4, and 7 dpi are shown in Fig. 4.

#### SiCon vs. SiFi2 Day 1 Sham

In order to examine the transcriptomic effect of SiFi2 in the uninjured spinal cord, GSEA was performed between sham groups. One day after sham surgery, SiFi2 administration led to 11 upregulated and 3 downregulated statistically significant pathways compared to SiCon (Fig. 4A). The upregulated pathways included immune responses (toll-like receptor signaling, hematopoietic cell lineage, cytokine-cytokine receptor interaction) and neuronal signaling (neuroactive ligand-receptor interaction). Common genes in these pathways were related to chemokine signaling (*Cxcl11*, *Cxcl10*, *Ccl20*, *Cxcl1*), pro- and anti-inflammatory cytokines (*Il6*, *Il11*, *Il1b*), and T cell activity (*Cd8a*, *Ciita*, *Rt1-Da*). There was also increased expression of genes related to neuronal receptors (*Drd1*, *Chrnb3*, *Gpr156*, *Htr6*, *Gabrr2*). The downregulated pathways included mitochondrial function (oxidative phosphorylation, Parkinson’s disease). Common genes among these pathways involved mitochondrial respiration (*Cox6a2*, *Cox4i2*, *Uqcrb*, NDUF family genes).

#### SiCon vs. SiFi2 Day 4 Sham

Four days after sham surgery, SiFi2 administration led to 5 upregulated statistically significant pathways compared to SiCon (Fig. 4B). The upregulated pathways included immune response pathways (natural killer-cell mediated cytotoxicity, Fc epsilon RI signaling, JAK/STAT signaling). Common genes in these pathways were related to Fc immunoreceptors (*Fcer1g*, *Fcgr3a*). Among the largest fold changes in these pathways were genes related to kininogen (*Kng1*), phospholipase A2 (*Pla2g2a*, *Pla2g5*), and the peripheral immune response (*Il2rb*, *Ncr3*, *Klrk1*).

#### SiCon vs. SiFi2 Day 7 Sham

Seven days after sham surgery, SiFi2 administration led to 6 upregulated and 2 downregulated statistically significant gene sets compared to SiCon (Fig. 4C). The upregulated pathways included immune responses (toll-like receptor signaling, natural killer cell-mediated cytotoxicity, cytokine-cytokine receptor interaction). Common genes in these pathways were chemokines (*Cxcl11*, *Cxcl9*) and immune cell receptor signaling (*Pik3r5*, *Vav1*). The downregulated pathways included mitochondrial function (oxidative phosphorylation, Parkinson’s disease). Common genes in these pathways involved mitochondrial respiration (*Cox6a2*, *Uqcrb*, and NDUF family genes).

#### SiCon vs. SiFi2 Day 1 Injury

In order to explore the transcriptomic effect of SiFi2 as a therapy, GSEA was performed at the same time points after SCI. At 1 dpi, SiFi2 administration led to 7 upregulated and 8 downregulated statistically significant pathways compared to SiCon after SCI (Fig. 4D). The upregulated pathways included those that intersect mitochondrial function and inflammation (oxidative phosphorylation, Parkinson’s disease, Alzheimer’s disease) and neuronal signaling (neuroactive ligand-receptor interaction). Common genes among these pathways were related to mitochondrial respiration [NADH: ubiquinone oxidoreductase core subunits (MT-ND and NDUF family genes)] and prostaglandin synthesis (*Cox1*,* Cox2*). The largest fold changes in these pathways involved pro-inflammatory cytokines (*Il1b*), neuronal receptors (*Chrnb4*,* Kiss1r*,* Chrna3*,* Gabra5*,* Trpv1*), and axon integrity/neuronal survival (*Uchl1*,* Bdnf*,* Atp1a3*). The downregulated pathways included peripheral immune responses (primary immunodeficiency, natural killer cell-mediated cytotoxicity) and cell division (cell cycle, homologous recombination). Common genes in these pathways were related to T cell activity (*Icos*,* Cd8a*), MHC antigen presentation (*Ciita*,* Rt1-M6-2*), and mitosis (*Pttg1*,* Espl1*,* Plk1*,* Ccnb1*,* Ccna2*). Some of the genes with more extreme negative fold changes were related to the peripheral immune response (*Siglec*,* Klrk1*).

#### SiCon vs. SiFi2 Day 4 Injury

At 4 dpi, SiFi2 administration led to 10 upregulated and 5 downregulated statistically significant gene sets compared to SiCon after SCI (Fig. 4E) The upregulated pathways included immune responses (cytokine-cytokine receptor interaction, hematopoietic cell lineage, chemokine signaling) and neuronal signaling (neuroactive ligand-receptor interaction). Common genes in these pathways were chemokine ligands (*Ccl20*,* Cxcl6*,* Ccl21*,* Cxcl13*,* Cxcl2*), cytokines (*Il6*,* Il11*,* Il2ra*), and growth factors (*Fgf7*). Other leading-edge upregulated genes were related to kininogen (*Kng1*), phospholipase A2 (*Pla2g2a*) and neuronal receptors (*Chrna3*,* Ntrk1*,* Trpv1*,* Cacng5*,* Chrnb3*). The downregulated pathways were primarily related to cell division (DNA replication, Cell cycle). Common genes in these pathways were involved in mitosis (*Prim2*,* Pttg1*,* Espl1*).

#### SiCon vs. SiFi2 Day 7 Injury

At 7 dpi, SiFi2 administration led to 37 upregulated and 5 downregulated statistically significant gene sets compared to SiCon after SCI (Fig. 4F). The upregulated pathways included cell adhesion (ECM receptor interaction, focal adhesion) and immune responses (hematopoietic cell lineage, cytokine-cytokine receptor interaction, JAK/STAT signaling). Common genes in these pathways were related to cell-to-matrix interactions (*Tnn*,* Thbs1*), phagocytosis (*Cd36*,* Cd68*), tissue remodeling (*Mmp9*,* Fn1*,* Lama2*,* Ctsk*,* Acp5*), TGF-β signaling (*Amh*,* Tgfbr2*,* Tgfbr1*,* Tgfb1*), peripheral immune cells (*Cd3e*,* Nfatc4*,* Cd8a*,* Cd8b*,* Ncf2*), integrins (*Itga2*,* Itgb2*,* Itga4*), cytokine signaling (*Il20rb*,* Il6*,* Il2rg*) and chemokine signaling (*Cxcl6*,* Ccl7*,* Cxcr4*). The secreted neuroregulatory protein *Spp1* was also among leading-edge genes. The downregulated pathways primarily included neuronal signaling (neuroactive ligand-receptor interaction, long-term potentiation, long-term depression) and calcium signaling (calcium signaling, cardiac muscle contraction). Common genes in these pathways were related to both neuronal receptors and calcium signaling (*Trhr*,* Htr2c*,* Grin1*,* Prkcg*,* Grm1*). Also among the top downregulated genes were those for neurofilaments (*Nefl*,* Nefh*,* Nefm*).

### Differential expression analysis

We also performed differential expression analysis to evaluate individual genes. The complete lists of differentially expressed genes identified after SCI alone are available in Supplementary Materials (Supplementary Tables S11–S13). No statistically significant genes were found between sham groups. Three differentially expressed genes were identified in comparisons between SCI + SiCon and SCI + SiFi2. SiFi2 significantly increased carbonic anhydrase 3 (*Car3*) (log2FC = 3.68, *p* adj = 0.02) at 1 dpi, increased prostaglandin-endoperoxidase synthase 2 or cyclooxygenase 2 (*Ptgs2*, *Cox2*) (log2FC = 1.23, *p* adj < 0.001) at 4 dpi, and decreased serine hydrolase like 2 (*Serhl2*) (log2FC = − 1.03, *p* adj = 0.04) at 4 dpi.

### Increased FL2 expression and knockdown in the lesion colocalizes with microglia/macrophages after SCI

**Fig. 5:**
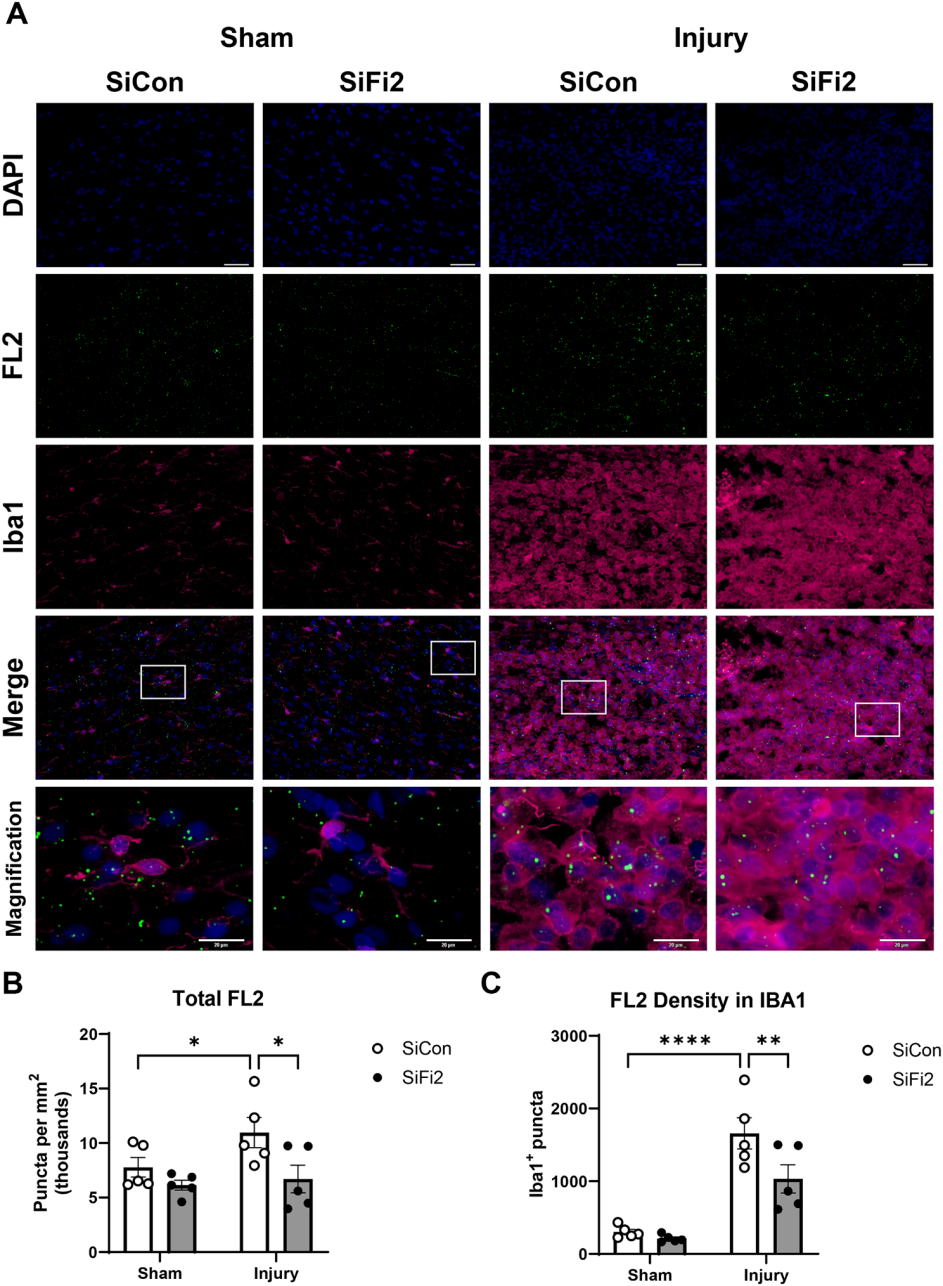
FL2 expression and microglia/macrophages visualized with dual in situ hybridization and immunohistochemistry after SCI. (**A**) Representative fluorescent images of dual in situ hybridization for FL2 (green) and Iba1 immunohistochemistry (magenta) with DAPI (blue) in the lesion at 4 dpi (20X; scale bar = 50 μm). High-magnification views show boxed regions of interest from merge images (scale bar = 20 μm). (**B**) Quantification of total FL2 as RNA puncta indicates a significant SCI-induced increase of FL2 and knockdown with FL2 siRNA (SiFi2) compared to control siRNA (SiCon). (**C**) Quantification of colocalized FL2 puncta in Iba1 indicates a significant SCI-induced increase of FL2 in microglia/macrophages and knockdown with SiFi2 compared to SiCon. *N* = 5/group. Two-way ANOVA with Šídák multiple comparison tests. *p < 0.05, **p < 0.01, ****p < 0.0001. Bars represent mean ± SEM

Since FL2 siRNA had significant effects on immune pathways, particularly at 4 dpi around the peak of the microglial response, we sought to further investigate SiFi2 and microglia/macrophages after SCI. Dual in situ hybridization and immunohistochemistry enabled us to quantify FL2 mRNA levels and assess localization with the protein Iba1 immunostaining for microglia and macrophages within the lesion (Fig. 5A). As previously shown in qPCR results at 1 dpi, SCI increased total FL2 expression in the lesion at 4 dpi, as shown between Sham + SiCon and SCI + SiCon (Fig. 5B, *p* = 0.049). Meanwhile, FL2 knockdown was confirmed between SCI + SiCon and SCI + SiFi2 (*p* = 0.01), demonstrating that SiFi2 administration prevented FL2 upregulation in the lesion site up to 4 dpi. SCI increased FL2 within Iba1^+^ regions at this time point (Fig. 5C, *p* < 0.0001). SiFi2 significantly reduced FL2 puncta in Iba1^+^ regions compared to SiCon (*p* = 0.008). Therefore, microglia and macrophages may be some of the cell types that upregulate FL2 after SCI.

### FL2 siRNA increases SCI-induced microglial accumulation

**Fig. 6:**
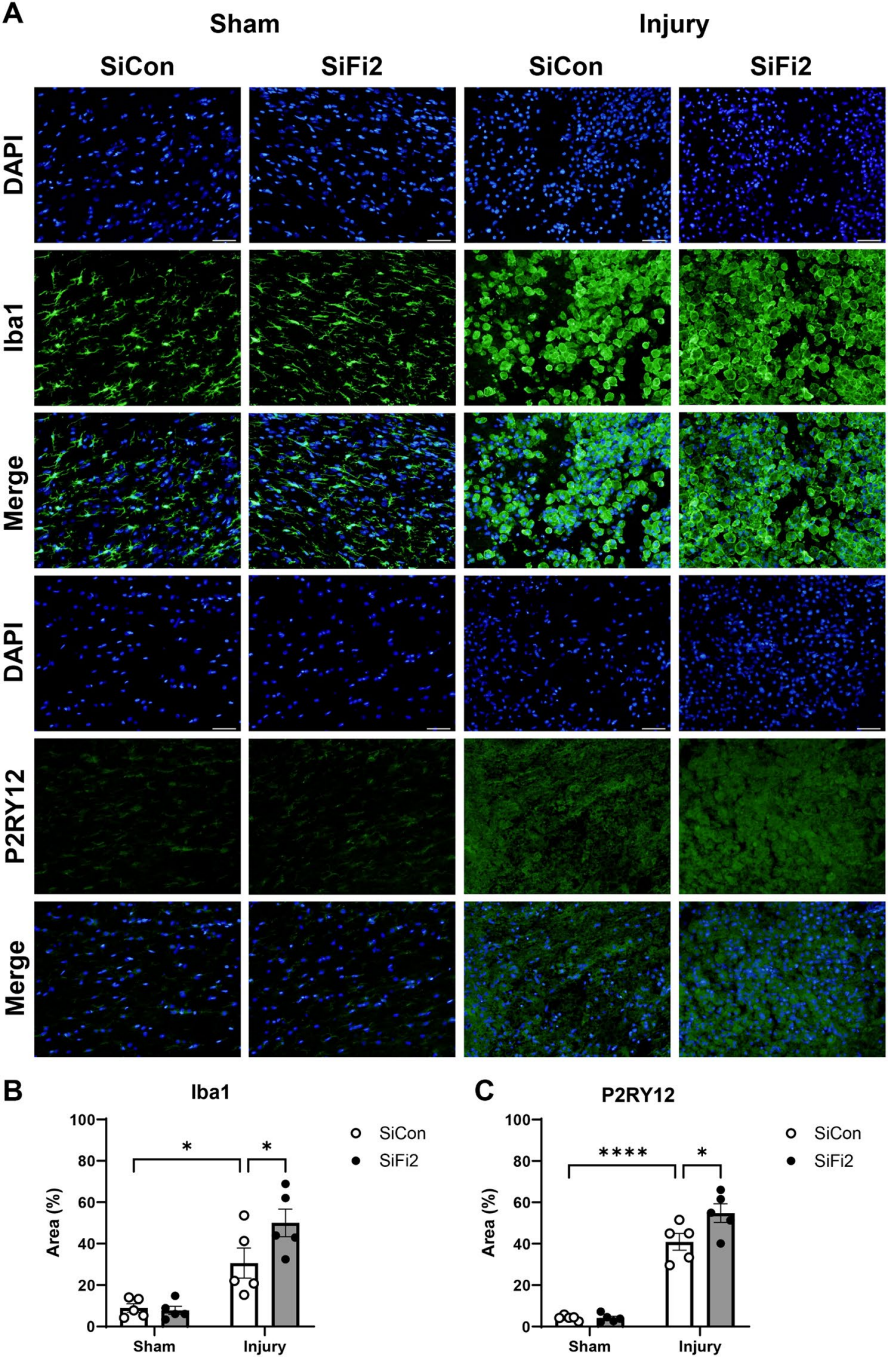
FL2 siRNA increases microglial accumulation in the lesion after SCI. (**A**) Representative immunofluorescence images of Iba1^+^ microglia/macrophages and P2RY12^+^ microglia cells (green) with DAPI (blue) in the lesion at 4 dpi after SCI and control siRNA (SiCon) or FL2 siRNA (SiFi2) treatment (20X; scale bar = 50 μm). Quantification of area occupied by Iba1^+^ microglia/macrophages (**B**) and P2RY12^+^ microglia (**C**) shows that SiFi2 increased microglia in the lesion site. *N* = 5/group. Two-way ANOVA with Šídák multiple comparison tests. *p < 0.05, ****p < 0.0001. Bars represent mean ± SEM

The accumulation of microglia and macrophages in the lesion or laminectomy site at 4 dpi was assessed with Iba1 and P2RY12 immunostaining (Fig. 6A). SCI + SiCon tissue showed significantly greater area occupied by Iba1^+^ microglia/macrophages as a percentage of area compared to Sham + SiCon (Fig. 6B, *p* = 0.02, two-way ANOVA with Šídák *post hoc* tests), demonstrating the recognized microglial/macrophage response to SCI. The percent area of Iba1^+^ microglia/macrophages was further increased by SiFi2 compared to SiCon after SCI (*p* = 0.03). P2RY12, which is more specific to microglia, also occupied a significantly greater percentage of the area after SCI + SiCon compared to Sham + SiCon (Fig. 6C, *p* < 0. 0001, two-way ANOVA with Šídák *post hoc* tests). The percent area of P2RY12^+^ microglia was further increased by SiFi2 compared to SiCon after SCI (*p* = 0.01). These results confirm that SiFi2 promotes the accumulation of microglia in the lesion after SCI.

### FL2 siRNA selectively increases acute neuroinflammation

**Fig. 7:**
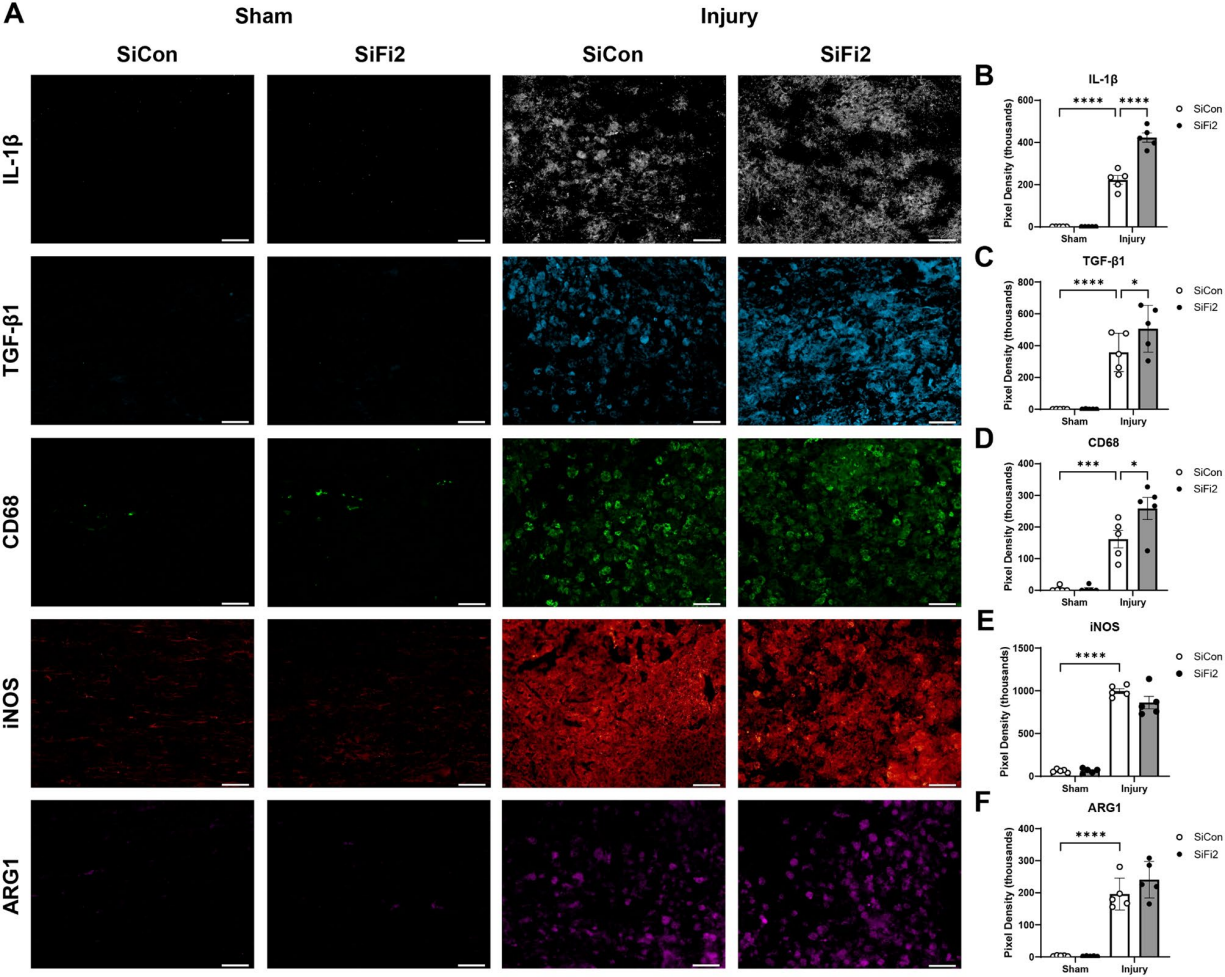
Inflammatory gene expression in the lesion after SCI and FL2 siRNA. (**A**) Representative immunofluorescence images of IL-1β (white), TGF-β1 (blue), CD68 (green), iNOS (red), and ARG1 (magenta) in the lesion 4 days after SCI or sham surgery and control siRNA (SiCon) or FL2 siRNA (SiFi2) treatment (20X; scale bar = 50 μm). IL-1β (**B**), TGF-β1 (**C**), and CD68 (**D**) after were significantly increased with SiFi2 compared SiCon after SCI. Neither iNOS (**E**) nor ARG1 (**F**) were significantly changed between treatments after SCI. *N* = 5/group. Quantification of Two-way ANOVA with Šídák multiple comparison tests. *p < 0.05, ***p < 0.001, ****p < 0.0001. Bars represent mean ± SEM

To determine if FL2 downregulation led to acute changes in major inflammatory proteins at the lesion site, immunohistochemistry was performed at 4 dpi (Fig. 7A). The immunostaining of all inflammatory markers was significantly increased after injury compared to sham. The sham-injured tissue showed little or no staining for any marker, and no significant difference was observed between sham treatment groups. Immunostaining for inflammatory cytokines showed significant increases after SCI + SiFi2 treatment compared to SiCon, including pro-inflammatory IL-1β (Fig. 7B, *p* < 0.0001, two-way ANOVA with Šídák *post hoc* tests) and anti-inflammatory TGF-β1 (Fig. 7C, *p* = 0.048, two-way ANOVA with Šídák *post hoc* tests). The phagocytic marker CD68 was also increased after SCI + SiFi2 compared to SiCon (Fig. 7D, *p* = 0.01, two-way ANOVA with Šídák *post hoc* tests). In contrast, the pro- and anti-inflammatory enzymes iNOS and ARG1 showed no significant difference between SiFi2 and SiCon after SCI. (Fig. 7E, F, two-way ANOVA with Šídák *post hoc* tests). These results indicated that SiFi2 selectively increased the expression of some proteins, both pro- and anti-inflammatory, in the lesion following SCI.

We tried to determine if FL2 downregulation altered acute lipid accumulation at the lesion site after SCI, as this pathological debris is typically cleared by microglia and macrophages. However, ORO staining showed no significant differences between SiFi2 and SiCon at 4 dpi (Supplementary Figure S3).

## Discussion

Therapies aimed at resolving inflammation and encouraging self-repair of CNS tissue are promising for the treatment of SCI. FL2 is a microtubule-severing enzyme previously shown to slow the rate of axon growth and wound healing [26, 28–30], but the use of FL2-targeted therapy in the context of CNS injuries has not been thoroughly investigated until now. In this study, we tested a novel approach for the treatment of SCI with nanoparticle-encapsulated FL2 siRNA. Our results demonstrated that acute FL2 knockdown promoted recovery in a moderate-severity contusion model of SCI. In the acute stage post-injury, FL2 knockdown increased both pro- and anti-inflammatory immune responses and promoted gene expression related to neuronal functions followed by improved functional and histological outcomes in the weeks following SCI.

While prior investigations found increased FL2 expression in the developing nervous system, particularly neurons [31], this is the first study to examine FL2 expression in the adult CNS and target FL2 expression after CNS injury. Given previous reports that FL2 reduces axon growth [28] and microglial functions [32], the localized upregulation of FL2 measured in the spinal cord lesion site and immediately rostral at 1 and 4 dpi suggests that increased FL2 after SCI may be detrimental to recovery. The data indicate that FL2 suppresses early spinal cord integrity and the acute immune response after CNS injury.

Administration of nanoparticle-encapsulated FL2 siRNA immediately after SCI was sufficient to improve locomotor function and restore hindlimb coordination exhibited in paw rotation. This supports our hypothesis that FL2 siRNA improves recovery after SCI and advances upon a prior study demonstrating FL2 siRNA improved regeneration and functional recovery after cavernous nerve injury [28]. The repeated functional analyses and the increase in significantly enriched gene pathways over acute time points, reaching 42 pathways at 7 dpi, indicate a large downstream effect of a single treatment administered at the time of injury. Whether this pattern and functional benefit is specific to treatment during acute SCI remains to be determined, but future research may determine the efficacy of delayed or sustained FL2 knockdown.

The effectiveness of microtubule stabilization in SCI recovery remains debated. Some studies report that microtubule-stabilizing drugs can reduce glial scarring, enhance axon regeneration, and improve functional recovery [38, 39]. However, others report that these drugs can impair regeneration and result in worse outcomes [40, 41]. While microtubule stabilization might prevent axon retraction and force growth, research indicates that increasing microtubule dynamic instability promotes intrinsic axon growth [42, 43]. Notably, dynamic instability increases in peripheral neurons after injury but does not in CNS neurons [42]. Our results align with these latter findings, suggesting that a reduction in FL2 and the subsequent increase in microtubule dynamic instability after SCI may increase the preservation or growth of CNS tissue.

The RNA sequencing and histological analyses confirm our prior research which suggested FL2 influences immune cell functions in vivo, particularly microglia and macrophages [32]. GSEA revealed evidence that FL2 siRNA significantly altered chemotactic and immune response gene expression pathways at all acute time points after both SCI and sham surgeries. After SCI, there were three different phases of the increased immune responses with FL2 knockdown: an immediate pro-inflammatory response (*Il1b*) at 1 dpi, a dual pro- and anti-inflammatory response (*Il6*, *Il11*) at 4 dpi, and an anti-inflammatory response (TGF-β signaling) at 7 dpi. Furthermore, there were concurrent increases in pro- and anti-inflammatory proteins (IL-1β, TGF-β1, and CD68) at 4 dpi. In combination with increased microglial accumulation at 4 dpi, the results support the hypothesis that FL2 siRNA increases SCI-induced neuroinflammation. Our findings also support prior research concluding that the acute microglial response may be beneficial to functional recovery. Moreover, this study substantiates the hypothesis that an increased neuroinflammatory response can lead to a stronger resolution of inflammation and prevent a “resolution plateau” [11, 20, 24, 25].

Importantly, by 28 dpi, we found a significant reduction in microglia/macrophages positive for Iba1 at the lesion site. These data suggest that the acute increase in inflammatory responses led to a more thorough resolution of injury. Several lines of research have suggested that the acute inflammatory response to SCI is dysfunctional, and that enhancing or altering it can improve future outcome [9, 10, 20–23]. The finding that FL2 downregulation can enhance the inflammatory response as measured by increases in Iba1, P2RY12, and both pro- and anti-inflammatory cytokines, suggests that this intervention is overcoming the dysfunctional inflammatory response and encouraging resolution.

Accompanying this, there was a significant increase in CC1 staining, suggesting an increase in oligodendrocyte presence after injury and treatment. Oligodendrocyte replenishment after SCI is important for functional recovery [44]. While we did not see any significant change in neuronal survival near the lesion, observations of improved functional recovery suggest that this increase in CC1 is a meaningful change. While the current study did not evaluate remyelination or regeneration, future analysis will pursue these lines.

The observed improvements in paw rotation are evidence of CST restoration [37], which is a major factor for voluntary motor functional recovery in both human and animal SCI. This finding is noteworthy given that SCI has been shown to induce atrophy and spontaneous sprouting of the CST that is further promoted with activity [45–47]. As previous studies have demonstrated, the increased *Il6* identified through GSEA may promote the regeneration of corticospinal and raphespinal tracts [48]. Given that reduced FL2 expression is associated with increased neurite growth even against an inhibitory environment and functional regeneration of peripheral nerves after injury [25], SiFi2 may have preserved CST integrity or promoted regeneration though our study did not test for regeneration.

This study also identified changes in neuronal gene expression associated with axon integrity and neurotransmission. GSEA detected increased expression of genes related to excitatory and inhibitory neurotransmission during the early time points that switched to reduced expression at 7 dpi. Although excitotoxicity may be harmful to neurons, it was recently reported that neurotransmitter phenotype switching occurs after adult rodent SCI where increased neurotransmission, particularly excitatory signaling, promotes sprouting and improves locomotor recovery [49]. Similarly, FL2 siRNA may initially promote early neurotransmitter phenotypes and activity-dependent axon integrity.

Differential expression analysis found that FL2 downregulation after SCI significantly altered the individual expression of the genes *Car3*, *Cox2*, and *Serhl2*, which have not previously been identified as associated with FL2. While *Car3* expression is upregulated after SCI, the significant increase of *Car3* at 1 dpi with FL2 siRNA may further protect cells from oxidative stress-induced cell death [50, 51]. The two significantly differentially expressed genes at 4 dpi, *Cox2* and *Serhl2*, are associated with immune responses. *Cox2* is upregulated after SCI to promote the immune response and inflammation [52, 53]. Less is understood about *Serhl2*, but downregulation of *Serhl2* after SCI has been found previously [54] and may be correlated with pathological peripheral immune cell infiltration [55]. While FL2 may directly impact pathways or functions related to these genes, it is more probable that these results are indicative of FL2 siRNA promoting cell survival and growth while also enhancing the resident immune response.

Comparisons between treatments in the sham groups revealed similarities and differences when juxtaposed to the analyses between injury groups. As observed after SCI, FL2 siRNA treatment in the sham animals also increased inflammatory gene pathways, further supporting the conclusion that FL2 regulates immune responses. However, these gene expression changes did not result in significant protein-level differences at 4 dpi in the sham groups. In contrast to SCI, FL2 siRNA treatment reduced gene pathways related to mitochondrial function in sham animals. This suggests an injury-specific response that makes it beneficial for treatment but may have a limited treatment profile. While the present study did not examine long-term outcomes of FL2 knockdown on sham animals, testing for potential negative effects of FL2 therapy may be of interest in consideration of CNS injury heterogeneity.

## Conclusion

These initial experiments are promising, showing that nanoparticle-encapsulated delivery of FL2 siRNA to the injured CNS achieved therapeutic benefits, and may be further optimized to improve outcomes of SCI. The results reveal a broader influence on immune responses than previously understood and support the hypothesis that FL2 knockdown increases acute pro-inflammatory and anti-inflammatory immune responses, thereby improving recovery after SCI. Findings also suggest that FL2 knockdown promotes long-term improvements, particularly in the CST. Future research will further test FL2 siRNA treatments and the translatability of FL2 therapy for SCI patients.

## Electronic supplementary material

Below is the link to the electronic supplementary material.

Supplementary Material 1

Supplementary Material 2

Supplementary Material 3

Supplementary Material 4

Supplementary Material 5

Supplementary Material 6

Supplementary Material 7

Supplementary Material 8

Supplementary Material 9

Supplementary Material 10

Supplementary Material 11

Supplementary Material 12

Supplementary Material 13

Supplementary Material 14

Supplementary Material 15

Supplementary Material 16

Supplementary Material 17

## Data Availability

The datasets generated and/or analyzed during the current study are available in the OSF repository, http://osf.io/du9zq.

## Abbreviations

BBB: Basso Beattie Bresnahan locomotor scale
CNS: Central Nervous System
DPI: Days Post-Injury
FL2: Fidgetin-like 2
GSEA: Gene Set Enrichment Analysis
KEGG: Kyoto Encyclopedia of Genes and Genomics (pathway or database)
ORO: Oil Red O
SCI: Spinal Cord Injury
SiCon: Control siRNA nanoparticles
SiFi2: FL2 siRNA nanoparticles
TMOS: Tetramethyl Orthosilicate
