# Supplementary figures and images for: Fidgetin-like 2 knockdown increases acute neuroinflammation and improves recovery in a rat model of spinal cord injury
Source: J Neuroinflammation. 2025 Mar 10;22:73. doi: 10.1186/s12974-025-03344-3 (PMC11895163; doi:10.1186/s12974-025-03344-3)

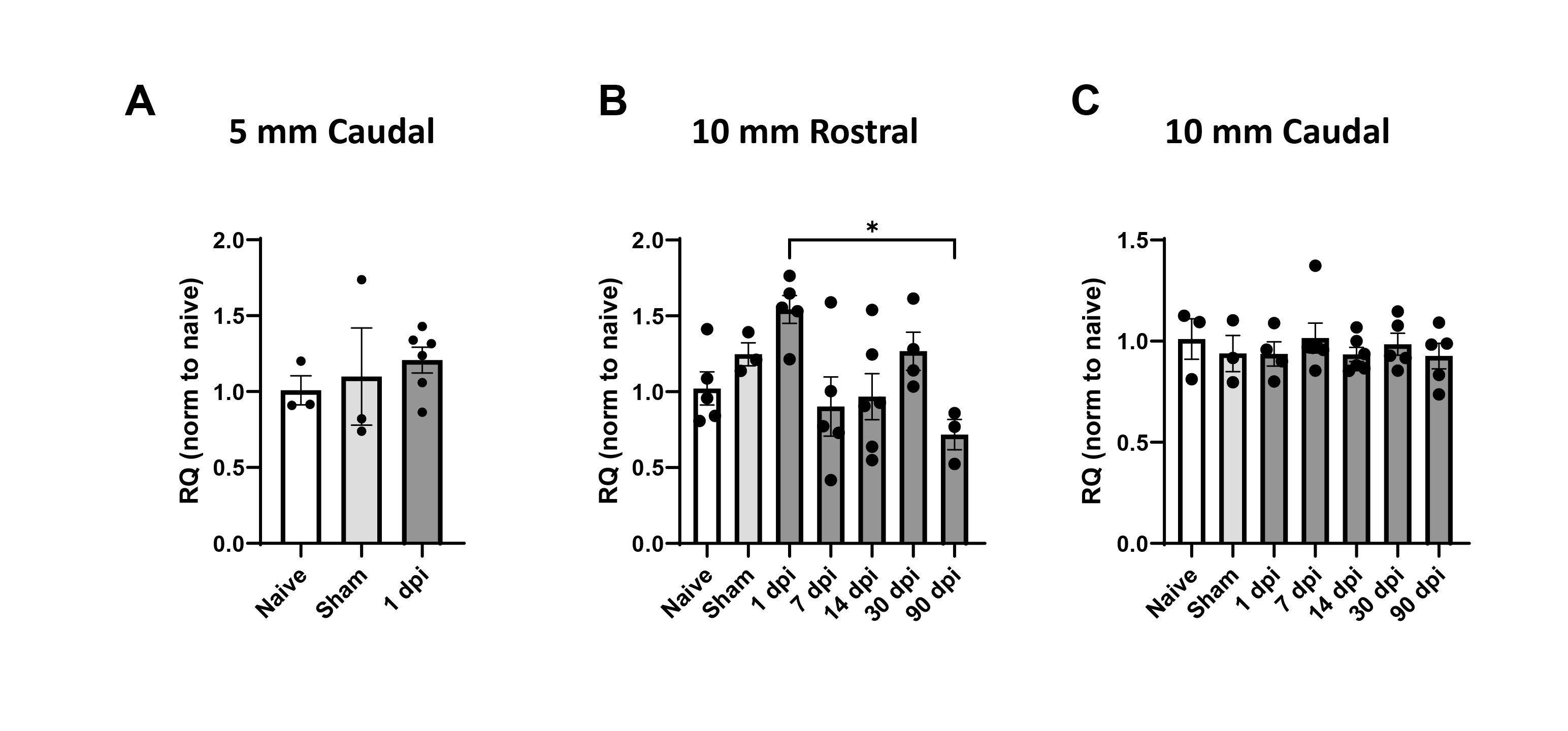

Supplement: Supplementary file 1 — Supplementary Material 1 [file 12974_2025_3344_MOESM1_ESM.tif]

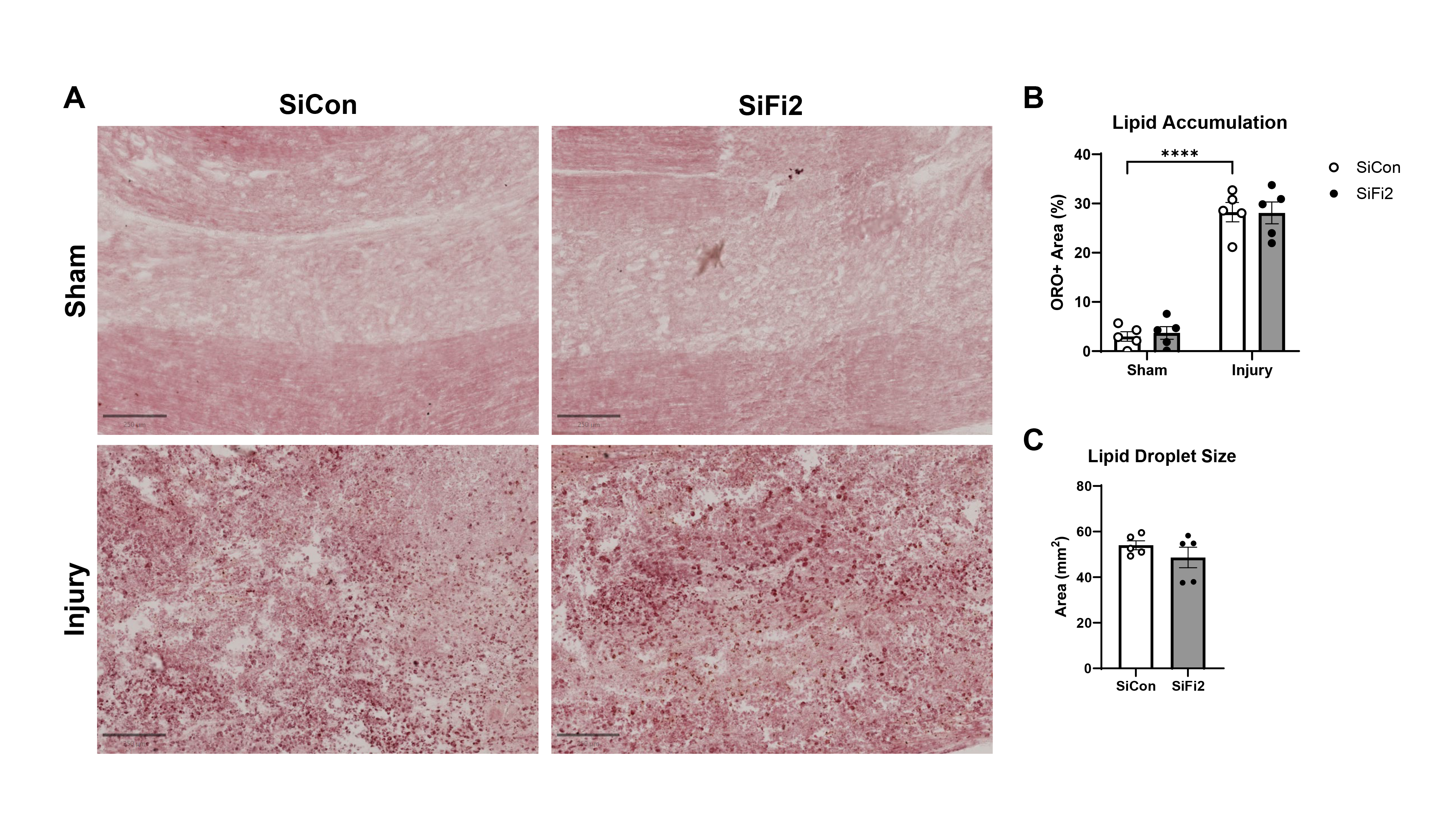

Supplement: Supplementary file 2 — Supplementary Material 2 [file 12974_2025_3344_MOESM2_ESM.tif]
