# Supplementary material for: Fidgetin-like 2 knockdown increases acute neuroinflammation and improves recovery in a rat model of spinal cord injury
Source: J Neuroinflammation. 2025 Mar 10;22:73. doi: 10.1186/s12974-025-03344-3 (PMC11895163; doi:10.1186/s12974-025-03344-3)
Supplement: Supplementary file 17 — Supplementary Material 17 [file 12974_2025_3344_MOESM17_ESM.docx]

**Supplementary Methods**

**Oil Red O**

A saturated solution of Oil Red O (ORO, Sigma-Aldrich, #O0625) was prepared at 0.5% in propylene glycol (Sigma-Aldrich, #P4347) and filtered to remove large particulates. Sections were immersed in 100% propylene glycol for 5 min at room temperature and then incubated in ORO Solution for 20 minutes at 60 °C. Then sections were immersed in 85% propylene glycol diluted in DI water and washed twice in 1x PBS. Slides were coverslipped with glycerol mounting medium (Abcam, #AB188804). Brightfield images were acquired using an Axioscan slide scanner (ZEISS) at 20X resolution. Images were analyzed using QuPath to quantify ORO stain vector area and average droplet size calculated as area divided by detected objects.

**Supplementary Figure S1** **FL2 mRNA levels distal to the lesion after SCI.** Relative quantities of *Fignl2* (FL2) mRNA at 5 mm caudal at 1 dpi (A), 10 mm rostral (B), and 10 mm caudal (C) to the lesion over time after contusion spinal cord injury. The samples were normalized to naive samples from 3 animals at the same spinal cord level. FL2 mRNA levels were quantified using the ddCT method and normalized to the housekeeping gene *Prkg1*. N = 3–6/group. Brown-Forsythe and Welch ANOVA with Dunnett’s multiple comparison tests. **p* < 0.05. Bars represent mean ± SEM.

**Supplementary Figure S2** **Gene set enrichment analysis (GSEA) of all genes in the injured spinal cord.** (A–C) Lollipop plots depicting statistically significant gene pathways identified in GSEA comparing sham + control siRNA (SiCon) and SCI + SiCon at 1, 4, and 7 dpi. The X axis shows the normalized enrichment score (NES), which indicates the degree to which genes in a particular set were upregulated (positive) or downregulated (negative) while accounting for the size of each gene set. The Y axis lists the individual KEGG pathways. The dot size is proportional to the total number of genes within each pathway (Set size). The dot color displays the range of adjusted *p* values < 0.05.

**Supplementary Figure S3 Acute lipid accumulation after SCI and FL2 siRNA**(A) Representative images of neutral lipid droplets by ORO in the laminectomy or lesion site at 4 dpi (AxioScan 10X resolution; scale bar = 250 μm). Quantitative analysis of ORO^+^ staining area (B) and average lipid droplet size (C) did not show a significant effect of SiFi2 on lipid acculumation at 4 dpi. N = 5/group. Two-way ANOVA with Šídák multiple comparison tests and unpaired *t*-test. *****p* < 0.0001. Bars represent mean ± SEM.
